# A Survey of Awareness of COVID-19 Knowledge, Willingness and Influencing Factors of COVID-19 Vaccination

**DOI:** 10.3390/vaccines10040524

**Published:** 2022-03-28

**Authors:** Juan Yang, Yuting Liao, Qianhui Hua, Huakun Lv

**Affiliations:** 1 State Key Laboratory of Molecular Vaccinology and Molecular Diagnostics, National Institute of Diagnostics and Vaccine Development in Infectious Diseases, School of Public Health, Xiamen University, Xiamen 361102, China; yangjuan661@163.com (J.Y.); liaoyuting@outlook.com (Y.L.); 2 School of Medicine, Ningbo University, Ningbo 315211, China; qhhua0426@outlook.com; 3 The Center for Disease Control and Prevention of Zhejiang Province, Hangzhou 310051, China

**Keywords:** COVID-19, SARS-CoV-2, knowledge, emergence use administration, vaccine acceptance, influencing factors

## Abstract

New vaccines are being developed in response to the coronavirus disease 2019 (COVID-19) pandemic. Vaccination provides a crucial preventive approach for managing COVID-19. We investigated adults’ willingness to take COVID-19 vaccines in the Zhejiang province, and their cognitions regarding COVID-19, when the COVID-19 vaccine is authorized under Emergency Use Administration. An online survey was conducted from September to October 2020, which included social-demographic characteristics, risk perception, acceptance and influencing factors in relation to COVID-19 vaccines. Multivariate logistic regression was performed to identify the influencing factors of vaccination acceptance. Of the participants, 70% intended to be vaccinated when the COVID-19 vaccine was approved under Emergency Use Administration, among 2171 valid questionnaires. Logistic regression revealed that being male, having a high cognitive score regarding COVID-19, the belief that the COVID-19 vaccine is safe and effective, and the belief that one will be infected with SARS-CoV-2 this fall and winter, were associated with a greater probability of accepting vaccination. Respondents with junior college/university education or above were less likely to accept vaccination. Concerns about the safety and effectiveness of the vaccine were the main factors hindering vaccination acceptance. Health education is important for promoting accurate public knowledge regarding COVID-19 vaccination.

## 1. Introduction

The coronavirus disease (COVID-19) pandemic has been a continuous global health threat since the first identification of the disease in December 2019, resulting in more than 418 million cumulative cases and 5 million deaths worldwide as of 18 February 2022 [1,2,3].

At present, there are no specific antiviral therapies for COVID-19, and vaccination against COVID-19 is considered to be one of the most cost-effective health interventions for the prevention and control of the pandemic [4,5,6]. Internationally, many countries are attempting to accelerate the research and development of COVID-19 vaccines. As of 18 February 2022, there have been more than 195 vaccines in pre-clinical development, with 144 vaccines in clinical development [7]. Currently, many types of COVID-19 vaccines, including mRNA, recombinant protein, adenovirus vector and inactivated virus vaccines have been approved in various countries. The results of clinical trials and real-world studies have demonstrated that the candidate vaccines are safe and effective [8]. However, with the development of the pandemic, inaccurate information about COVID-19, such as information underestimating the severity of the pandemic or ignoring the detrimental effects of the pandemic, has become widespread [9]. Meanwhile, there have been numerous reports of problems after vaccination published on the Internet, such as muscle soreness, fever and even immune system diseases [10]. These findings have led to increasing public doubts about the safety and reliability of the vaccine, all of which may affect expectations regarding vaccination [11]. Vaccine hesitancy may be responsible for the low COVID-19 vaccination rate. The Strategic Advisory Group of Experts (SAGE) defined vaccine hesitancy as a “delay in acceptance or refusal of vaccines despite availability of vaccination services” [12]. Communication and media environments are potential drivers of vaccine hesitancy [13]. Social media is connecting people while rapidly spreading, sharing and acquiring large-scale knowledge about COVID-19. This knowledge may increase awareness of COVID-19 vaccines, shed light on their efficacy and safety and reduce vaccine hesitancy. However, social media has created an “infodemic”, an overload of information both online and offline, where too much information (some of it right, some of it wrong) makes it hard to find reliable sources [14]. False information and rumors about COVID-19 vaccination have emerged on social media, with people unable to tell whether the information is real or not, leading to people hesitating about vaccination.

Thus, to control COVID-19, it is very important to understand the public’s willingness to vaccinate and the associated factors, which can serve as an early warning system to prompt necessary measures to prevent the decline of vaccine acceptance and trust. Many studies have begun to focus on the factors that may affect people’s willingness to be vaccinated. For example, Robinson and colleagues found that younger, less educated, lower-income and ethnic minority populations were less likely to be vaccinated [15]. Taylor et al. [16] reported that vaccination attitudes are closely related to cognitions regarding the pandemic, such as the perceived risk of COVID-19 and the perceived severity of SARS-CoV-2. Qian Zhou et al. [17] reported that risk perception regarding the COVID-19 pandemic was significantly associated with acceptance of vaccination. Therefore, the current study aimed to understand cognitions regarding COVID-19 and SARS-CoV-2, the relationship between them and to identify factors causing vaccine hesitancy or acceptance. Clarifying this issue is critical for updating vaccination strategies and immunization programs against COVID-19 in future.

## 2. Materials and Methods

### 2.1. Study Design, Population and Sampling

A cross-sectional online survey was conducted using convenience sampling and snowball sampling strategies from September to October 2020. The survey was conducted online using the largest online survey platform in China: Wen Juan Xing. This platform is equivalent to Qualtrics and SurveyMonkey and provides online questionnaire design and survey functions for enterprises, research institutions and individuals. Participants aged over 18 could fill in the questionnaire anonymously. The online survey link was disseminated via QQ (https://im.qq.com/index accessed on 18 February 2022) and WeChat (https://weixin.qq.com/ accessed on 18 February 2022), on which personal information and public websites can be shared with family members, friends and colleagues and forwarded to others by participants. According to the sample size formula $n={\left(\frac{Z_{\left(1-\frac{\alpha }{2}\right)}}{d}\right)}^{2}p\left(1-p\right)\;\times \;Deff$ required for the cross-sectional investigation, the willingness rate of COVID-19 vaccine vaccination reported in literature *p* ≈ 80% was taken, as well as the significance level *α* = 0.05 and the absolute allowable error *d* = 2.5%. This survey used non-random sampling, where *Deff* = 2 was taken as the design effect. Considering the sample loss caused by unpredictable factors, the sample size was increased by about 10% on the basis of the above estimated sample size, and the minimum sample size required was calculated as *n* = 2164. In order to ensure the validity of online questionnaires, 2171 valid questionnaires were obtained by excluding 12 invalid questionnaires that did not meet the requirements.

### 2.2. Measures

In the current study, the questionnaire was designed by consulting domestic and foreign literature on vaccination intention. We referred to well-studied questionnaires with a high degree of credibility and validity in the relevant literature, combined with information related to COVID-19 infection, the COVID-19 vaccine and the social and cultural background in China. The questionnaire included the following contents: socio-demographic characteristics, knowledge of COVID-19, risk perception of COVID-19, willingness regarding emergency vaccination, the COVID-19 vaccine, views on the safety and protective effects of the COVID-19 vaccine and the factors affecting vaccination acceptance. To clarify potential problems with this questionnaire, several small sample pre-surveys were conducted before the formal survey, and the questionnaire was modified and improved on the basis of the results of returned questionnaires and any shortcomings identified via respondents’ feedback.

### 2.3. Questionnaire

The variables measured in this study included socio-demographic characteristics, such as gender, age, residence, education level, monthly income and other general demographic variables. Knowledge about COVID-19 was divided into four dimensions: COVID-19 related knowledge, with 10 items; transmission mode of COVID-19, with seven items; symptoms after COVID-19 infection, with 8 items; and preventive measures for COVID-19 infection, with 9 items. Respondents rated the statements as “true”, “false”, or “unclear”. Risk perception of COVID-19 was examined with two questions: “Do you think the domestic COVID-19 epidemic will break out again in autumn and winter this year” and “Do you think you will be infected with COVID-19 this autumn and winter”. Each question was scored on a scale of 0–10 based on the probability perceived by the sample population, with 0 indicating impossible and 10 indicating very likely. The higher the score, the higher the perceived risk of COVID-19. Opinions on the safety and protective effects of COVID-19 vaccines were assessed with two questions: “What is your opinion regarding the safety of COVID-19 vaccines currently entering phase III clinical trials in China?” and “What is your opinion regarding the protective effects of COVID-19 vaccines currently entering phase III clinical trials in China?” Respondents answered using a scale of 0 to 100, with 0 indicating unsafe or ineffective, and 100 indicating very safe or effective. The Likert Scale 6 was adopted for assessing respondents’ willingness to receive the COVID-19 vaccine for emergency use, and the score range was 0–10, with 0–1 indicating definitely unwilling, 2–3 indicating probably unwilling, 4–5 indicating somewhat unwilling, 6–7 indicating somewhat willing, 8–9 indicating willing and 10 indicating definitely willing.

### 2.4. Statistical Analysis

Microsoft Excel 2019 software was used to organize the data, and SPSS 21.0 software was used for statistical analysis. We used the following descriptive analysis procedure: (1) describing the general demographic characteristics of the respondents; (2) describing the scores of respondents’ knowledge and opinions on COVID-19, scoring the four dimensions, respectively; awarding points for correct answers and not for incorrect or “don’t know” answers, and converting each of the four dimensions to a score of 10; (3) views on the safety and protective effects of the COVID-19 vaccine were transformed to a 10-point system. We described the willingness of emergency vaccination in terms of proportion and frequency; scores of 0–5 were combined with willing vaccination, and scores of 6–10 were combined with unwilling vaccination. The influencing factors of vaccination intention were analyzed using chi-square tests. After univariate analysis, multivariate logistic regression (Forward: LR) was used to conduct multivariate analysis to further explore the influencing factors of the change of COVID-19 vaccine vaccination intention and mitigate the impact of confounding factors.

In this analysis, the inclusion criterion was α = 0.05, and the exclusion criterion was α = 0.10. The odds ratio (OR) and 95% confidence interval (CI) for “willing” versus “unwilling” were calculated. The test level was α = 0.05.

## 3. Results

### 3.1. Study Sample Characteristics

A total of 2171 valid questionnaires were included in our analysis. The respondents were from all 11 cities in Zhejiang Province. Respondents’ basic information included gender, age, residence, education, monthly income, and other demographic characteristics. The male to female ratio was 1:1.53. The proportions of respondents aged 18–30 years old, 31–40 years old, 41–50 years old and ≥51 years old were 29.4%, 32.2%, 24.3% and 14.1%, respectively. Respondents living in urban and suburban/rural areas accounted for 75.2% and 24.8% of the sample, respectively. The proportions of respondents who had completed technical secondary school or below, junior college/university, or a master’s degree or above were 11.7%, 71.8% and 16.5%, respectively. Respondents with monthly incomes of CNY < 3000, CNY 3000–5000, CNY 5000–10,000 and CNY > 10,000 accounted for 13.4%, 24.1%, 38.7% and 23.8% of the sample, respectively (Table 1).

### 3.2. Knowledge of COVID-19

Knowledge about COVID-19, including COVID-19 related knowledge, the mechanism of transmission, symptoms or manifestations of COVID-19 infection, and preventive measures for COVID-19 infection, were significantly associated with respondents’ accurate understanding of COVID-19. Knowledge about COVID-19 was divided into four dimensions, and respondents judged the statements of each item as “true”, “false” or “unclear”.

#### 3.2.1. The First Part of the COVID-19 Related Knowledge Consisted of Ten Items

According to the survey results, the correct answer rates for the items “COVID-19 can not spread among people easily”, “COVID-19 is not a serious public health problem” and “There are specific drugs for the treatment of COVID-19” were relatively low, accounting for 1.38%, 3.59% and 7.55% of responses, respectively. The correct answer rates for the items “COVID-19 is an acute viral infection”, “COVID-19 is spreading worldwide and can infect anyone in any age”, “COVID-19 is a serious disease that can cause death”, “COVID-19 infection is more serious and has a higher mortality rate in older people” and “Contracting COVID-19 is more severe for people with chronic diseases, with higher mortality rates”, were relatively high, as all the accuracy rates were over 90% (Table 2) and the accuracy of the remaining two items was 81.85%.

#### 3.2.2. The Second Component Was the Mode of Transmission of COVID-19, with Seven Items

There were five items with a judgment accuracy of more than 85%: “transmission by droplets from an infected person”, “touching elevators, tables, door handles, handrails, coins, or paper money”, “exposure to fecal contaminants”, “frozen food imported from abroad” and “aerosols”. A total of 79.92% of the respondents correctly judged that “shaking hands with infected persons” was a means of spreading SARS-CoV-2. Only 51.08% of the respondents correctly responded that “contact with pets” could spread SARS-CoV-2 (Table 3). The results revealed that for the common and widespread transmission mode of SARS-CoV-2, such as droplet transmission, the respondents had a high level of accuracy and clear understanding. However, evidence that indirect contact, such as “touching the same doorknob, handrail or other items with an infected person” or “shaking hands with an infected person”, can also spread COVID-19, was misunderstood by many respondents.

Only 330 respondents correctly responded to all 7 items of this dimension, accounting for 15.20% of the sample (Table 3).

#### 3.2.3. The Section of the Questionnaire Regarding the Symptoms or Manifestations of COVID-19 Infection Contained Eight Items

More than 90% of respondents judged correctly that SARS-CoV-2 infection can lead to “fever”, “cough”, “fatigue”, “pharyngalgia”, “myalgia” and “dyspnea”. A total of 85.17% and 87.98% of respondents correctly responded that “rhinobyon/running nose” and “vomiting/diarrhea” were symptoms of SARS-CoV-2 infection, respectively (Table 4). A total of 77.34% of respondents correctly judged all eight items of this dimension.

#### 3.2.4. The Fourth Part of the Questionnaire on SARS-CoV-2 Infection Prevention Measures Contained Nine Items

More than 98% of respondents correctly responded regarding whether measures such as “frequent hand-washing”, “keeping an appropriate distance from other people”, “avoiding rubbing the eyes, mouth and nose”, “wearing masks in public places”, “avoiding touching elevator buttons, door handles, and other surfaces in public facilities” and “ventilating the room” could prevent COVID-19. The accuracy of responses regarding whether “eating garlic” and “taking antibiotics” can effectively prevent COVID-19 was low, accounting for 17.55% and 14.37% of responses, respectively. A total of 55.69% of respondents answered all nine questions correctly (Table 5).

### 3.3. Analysis of COVID-19 Vaccination Willingness and Influencing Factors

#### 3.3.1. Frequency Distribution and Chi-Squared Analysis of Intentions of COVID-19 Vaccination

The score of COVID-19 knowledge, transmission, infection symptoms and preventive measures was divided into three parts [0, 8), [8, 9) and [9, 10) for univariate analysis, the high score group” [9, 10)” including score of 9 and 10, a score of 8 was included in the intermediate group” [8, 9)”, the low score group” [0, 8)” including a score of 0. Among the 2171 respondents in the emergency use of COVID-19 vaccine, 1517 reported that they were willing to be vaccinated, accounting for 70% of the sample. Univariate analysis revealed that vaccination intention rates among men and women were 76.8% and 65.4%, respectively. Men were significantly more willing to receive the vaccination (*χ*^2^ = 32.06, *p* < 0.001). Older age was associated with greater willingness to vaccinate (*χ*^2^ = 26.76, *p* < 0.001). Respondents who were ≥51 years old had the highest rate of vaccination willingness (79.7%). The rates of vaccination willingness among respondents with technical secondary school education or below, college/university education and a master’s degree or above were 85.8%, 69.0% and 62.4%, respectively. Thus, the rate of vaccination willingness among respondents with a higher level of education was lower than that among respondents with a lower level of education (*χ*^2^ = 40.454, *p* < 0.001). Respondents whose monthly income was CNY 3001–5000 or CNY 5001–10,000 had high vaccination willingness (*χ*^2^ = 10.876, *p* = 0.012). In addition, the higher the level of COVID-19 related knowledge, the higher the willingness to vaccinate (*χ*^2^ = 15.47, *p* < 0.001). However, “scores of COVID-19 transmission”, “scores of COVID-19 infection symptoms” and “scores of COVID-19 prevention measures” were not significantly different (*p* > 0.05). Respondents who believed that COVID-19 vaccines were safe (88.7%) said they would get vaccinated (*χ*^2^ = 291.82, *p* < 0.001). A total of 89.6% of respondents who believed that COVID-19 vaccines were effective said they would get vaccinated, and 54.2% of respondents who thought that COVID-19 vaccines were ineffective said they would get vaccinated; the former group had a higher rate of vaccination intention (*χ*^2^ = 276.41, *p* < 0.001) (Table 6).

Thus, the results revealed that gender, age, education level, monthly income, COVID-19-related knowledge level, COVID-19 risk awareness (“Do you think the domestic COVID-19 epidemic will break out again” or “you will be infected with SARS-CoV-2 in autumn and winter this year?”) and views on the safety and effectiveness of COVID-19 vaccine were significant factors affecting the respondents’ willingness to receive the COVID-19 vaccine in a public health emergency.

#### 3.3.2. Factors Associated with Willingness to Receive the COVID-19 Vaccine

The variables that reached significance in the univariate analysis were included in the multivariate logistic regression analysis: Men were more willing to accept vaccination (OR = 1.54; 95% CI: 1.24–1.92) than women. People who have a high score in COVID-19-related knowledge were more likely to be vaccinated (OR = 1.46, 95% CI: 1.06–2.00) with COVID-19 than those with a lower score. Respondents who believed that the COVID-19 vaccine was safe (OR = 2.56, 95% CI: 1.68–3.92) and effective (OR = 3.06, 95% CI: 1.99–4.71) were associated with a greater willingness to be vaccinated. Those who reported concerns about becoming infected with COVID-19 this fall and winter (OR = 2.18, 95% CI: 1.27–3.77) were more likely to be willing to be vaccinated compared with those who had no concerns of becoming infected. Respondents with an education level of junior college/university (OR = 0.45, 95% CI: 0.30–0.68) or a master’s degree or above (OR = 0.35, 95% CI: 0.22–0.56) were less likely to accept vaccination compared to respondents with technical secondary school and below (Table 7).

#### 3.3.3. Reasons for COVID-19 Vaccination

Respondents who were willing to get vaccinated and agreed (“agree” and “strongly agree”) with the statements “COVID-19 vaccination is safe”, “Vaccination is very effective for preventing COVID-19”, “COVID-19 vaccination can protect my family/friends/colleagues from infection” and “Vaccination is beneficial if it is recommended by the government” accounted for 83.42%, 83.73%, 86.44% and 91.06%. Respondents who were unwilling to receive the COVID-19 vaccine and agreed or strongly agreed that “There are no COVID-19 cases in the region and no vaccinations are required”, “The effectiveness of the COVID-19 vaccine is doubtful”, “The side effects of the COVID-19 vaccine are worrying” and “Community health service centers are not convenient for vaccination” accounted for 31.51%, 39.89%, 60.65% and 12.75% of the sample, respectively. Concerns about vaccine effectiveness (39.89%) and side effects (60.65%) were the main reasons for the respondents’ reluctance (Table 8).

## 4. Discussion

The survey in the current study adopted the Likert Scale 6, on the basis of recommended scoring methods for examining vaccine hesitancy, unlike other surveys that used “willing”, “uncertain” or “unwilling” for classification selection. The data obtained using the Likert Scale were likely to be closer to the real willingness to receive the COVID-19 vaccine following authorization under Emergency Use Administration (EUA) [18]. According to the current survey results, respondents who were definitely unwilling, probably unwilling, somewhat unwilling, somewhat willing, willing and definitely willing to be vaccinated accounted for 9%, 6.8%, 14.4%, 14.8%, 20.1% and 35% of the sample, respectively. Respondents who were willing (somewhat willing, willing and definitely willing) to take the vaccine accounted for 70% of the sample, which was slightly higher than the rate of vaccination intention (68%) reported by Nguyen et al. [19], and approached the rate reported in a global study in which 71.5% of respondents reported that they would take a vaccine if it were proven to be safe and effective [20]. The current results indicated that respondents who recognized the safety and protective effects of the COVID-19 vaccine were more willing to get vaccinated (*p* < 0.001). The main reasons for respondents’ reluctance to get vaccinated were concerns about the efficacy of the vaccine and its potential side effects. Thus, the efficacy and safety of the vaccine were the main factors influencing vaccination willingness, which is consistent with the findings of previous studies [20,21,22]. An absence of concerns about vaccine safety and increased awareness regarding vaccine side effects have been reported to make people more likely to take the vaccine [18,23].

The univariate analysis results revealed that men, older people, those with a low education level, those with a high COVID-19-related knowledge level and those with a high COVID-19 risk awareness level had a higher rate of vaccination willingness (*p* < 0.001). The current results revealed that the older the respondents were, the higher their willingness to be vaccinated against COVID-19, which was consistent with the findings reported by Lazarus et al. [20]. Compared with younger age groups, older people face a greater risk from various pathogenic microorganisms because of the deterioration of bodily function and poor immune function. For infectious pathogens, older people are more likely to experience serious illness, with higher case fatality rates [24]. Because people in older age groups are more susceptible to serious COVID-19 infections and death, an increased fear of disease in this group has been reported to lead to favorable attitudes toward COVID-19 vaccines [22]. In addition, because older people tend to receive vaccine information through official channels such as television and radio, they are less likely to be exposed to false news, contributing to the higher likelihood of vaccine acceptance. The current results revealed that men exhibited a higher rate of willingness to be vaccinated against COVID-19 compared with women, which is consistent with previous studies reporting higher rates of influenza vaccination among men [25,26]. This may be because men have a higher perception of disease risk compared with women. A previous study reported that the case fatality rate of COVID-19 in men (4.7%) was higher than that in women (2.8%) [27]. We found that highly educated groups were significantly less willing to be vaccinated for emergency use of COVID-19 vaccines. This may be because more educated individuals receive more information from social networks and various channels and have greater concerns about the effectiveness and side effects of COVID-19 vaccines, affecting their willingness to be vaccinated. Some previous studies of influenza vaccination reported similar results [28,29]. On the basis of the transmission characteristics of SARS-CoV-2, the public’s awareness of COVID-19 is important for the prevention of disease and control of the epidemic. Investigation of knowledge and attitudes related to COVID-19 can be helpful for clarifying the level of public awareness, which can inform approaches for the prevention and control of the epidemic. Many cognitive factors contribute to people’s health protection behavior during epidemics, including risk cognition and susceptibility cognition. Other cognitive factors, including the accurate understanding of the mode of virus transmission and cognitions regarding the behavior for effectively reducing the risk of infection, will also have a positive impact on people’s health protection behavior. On the contrary, unclear information and negative attitudes may lead to pain and panic during an epidemic [30,31]. The current survey findings revealed that the respondents had substantial knowledge about COVID-19, but the level of accuracy regarding the transmission route and prevention measures of COVID-19 was relatively low. A high percentage of respondents incorrectly believed that there are specific drugs for treating COVID-19. Respondents had relatively high accuracy in judging the more common modes of transmission of SARS-CoV-2, but a low level of awareness of evidence that indirect contact can also transmit COVID-19. The accuracy rate for judging whether “eating garlic” and “taking antibiotics” can effectively prevent COVID-19 was low. A previous survey conducted in 19 countries reported that knowledge significantly affects precautionary measures through the effectiveness of belief, and had a direct effect on attitudes [32]. The current results also revealed that respondents with high risk awareness exhibited greater willingness to be vaccinated, which is consistent with a previous study [33]. Another study reported that a high level of knowledge was significantly associated with more positive attitudes and perceptions [34]. Thus, it is important to raise public awareness of COVID-19.

Emerging evidence suggests that both exposure to misinformation about COVID-19 [9,35] and public concern regarding the safety of vaccines may be contributing to the observed decline in the intention to be vaccinated [36]. This highlights the need for measures to address public acceptability, trust and concern over the safety and benefits of approved vaccines [37].

Media platforms should actively fulfill their public responsibilities, carry out science popularization in a targeted way and enable scientific and rational voices to guide the public to raise awareness about COVID-19. However, the authenticity and effectiveness of information disseminated by various we-media should be guaranteed, and the leakage of false and inflammatory information should be avoided. Media platforms can set up targeted publicity campaigns for different groups in the population to promote their access to health knowledge and create knowledge bases of different depths to meet the needs of different groups. The dangers of SARS-CoV-2 and the need for vaccination should be actively promoted during vaccine promotion efforts. Confirmation of the effectiveness of the vaccine by authoritative sources, advice from medical staff, and promotion of the effectiveness of the vaccine by official media can all motivate the public to choose vaccination [32]. After the vaccine is available on the market, government departments and relevant media should publish vaccine information scientifically and objectively, alongside professional advice from the Center for Disease Control and medical staff, which will improve the public’s confidence in vaccines and their willingness to get vaccinated against COVID-19. To increase the urgency of vaccination and enhance the awareness of the necessity of COVID-19 vaccination, media platforms should actively guide and educate the public. The government is an important factor in the awareness and practice of COVID-19 vaccination, and its attitude towards COVID-19 vaccination has a direct impact on the vaccination rate of the public. The government should disseminate accurate information through health publicity and education to promote rational understanding of vaccines, and to actively promote vaccination.

The current survey involved several limitations. Convenience sampling was adopted in the study, but random sampling was not performed, potentially affecting the representativeness of the study sample. Due to the fact that the participants in our study came from an area that was not affected severely by COVID-19, the findings in this study are not generalizable for residents who live in other areas in China. Future studies should recruit a more representative and larger participant pool. The network questionnaire survey was self-reported and may involve some information bias. This questionnaire was administered from September to October 2020, at which time the vaccination policy was markedly different from the current vaccination policy. At present, the nationwide free vaccination campaign in China is progressing steadily, and most COVID-19 vaccines have been conditionally marketed. Because our study assessed respondents’ willingness to be vaccinated when the vaccine is authorized under EUA, the results may not reflect the true willingness to be vaccinated after marketing. In the context of changing global epidemics and clinical advances in COVID-19 vaccines, public perception and demand for COVID-19 vaccines are also changing. Thus, it will be necessary to investigate the public’s willingness to receive vaccinations at different periods during the COVID-19 pandemic.

## 5. Conclusions

The current results revealed that 70% of adult respondents reported a willingness to receive the COVID-19 vaccine once it is authorized under EUA in China during the pandemic period. In addition, the results revealed that males, older people, those with a high education level, those with high awareness of COVID-19, those with high awareness of COVID-19-related risks and those who recognized the safety and efficacy of the COVID-19 vaccine were more willing to get vaccinated. In the survey, the respondents’ correct rate of judgment on the transmission mode and prevention measures of COVID-19 was low. Different media platforms, government agencies, and institutions should actively carry out targeted promotion of science, publicize the dangers of COVID-19 and the necessity of vaccination and improve the public’s awareness of COVID-19, to encourage the public to get vaccinated against COVID-19.

## Figures and Tables

**Table 1:** Demographic characteristics of participants.

Items	Respondents (*n* = 2171) N (%)
Gender	
Male	857 (39.5)
Female	1314 (60.5)
Age group	
18–30	639(29.4)
31–40	698(32.2)
41–50	528 (24.3)
≥51	306 (14.1)
Region	
Urban	1632 (75.2)
Rural	539 (24.8)
Highest level of education	
Technical secondary school and below	253 (11.7)
Junior College/University	1559 (71.8)
Master’s degree or above	359 (16.5)
Monthly income/CNY	
≤3000	290 (13.4)
3001–5000	524 (24.1)
5001–10,000	840 (38.7)
>10,000	517 (23.8)

**Table 2:** Participants’ responses to COVID-19 related knowledge.

Items	Ture/%	False/%	Unclear/%
COVID-19 is an acute viral infection	91.20	4.88	3.92
COVID-19 is spreading worldwide and can infect anyone in any age	99.12	0.23	0.64
COVID-19 can not spread among people easily	1.38	97.88	0.74
COVID-19 is a serious disease that can cause death	94.10	3.04	2.86
COVID-19 is not a serious public health problem	3.59	94.01	2.40
COVID-19 affects the economy by reducing labor productivity and increasing the burden of healthcare costs	81.85	11.42	6.73
COVID-19 infection is more serious and has a higher mortality rate in older people	92.95	2.99	4.05
Contracting COVID-19 is more severe for people with chronic diseases, with higher mortality rates	92.03	2.16	5.80
There are specific drugs for the treatment of COVID-19	7.55	73.19	19.25
Vaccination is the most effective way to prevent COVID-19 and its complications	81.85	5.53	12.62

**Table 3:** Participants’ responses to the mode of transmission of COVID-19.

Propagation Mode of COVID-19	True/%	False/%	Unclear/%
Transmission by droplets from an infected person (talking, coughing, sneezing)	99.59	0.18	0.23
Shaking hands with an infected person	79.92	16.90	3.18
Touch elevators, tables, door handles, handrails, coins or paper money	88.95	7.88	3.18
Contact pets	51.08	33.44	15.48
Exposure to faecal contaminants (public toilets)	87.47	7.23	5.30
Frozen food imported from abroad (seafood, meat)	86.27	6.77	6.96
Aerosols (aerosols formed when droplets are mixed in the air and can cause infection when inhaled)	95.49	1.80	2.72

**Table 4:** Participants’ responses to symptoms and manifestations of COVID-19 infection.

COVID-19 Infection Symptoms or Manifestations	True/%	False/%	Unclear/%
Fever	98.85	0.41	0.74
Cough	97.60	0.78	1.61
Fatigue	98.11	0.60	1.29
Rhinobyon/running nose	85.17	7.51	7.32
Pharyngalgia	92.49	2.90	4.61
Myalgia	92.40	3.27	4.33
Vomiting/diarrhea	87.98	5.11	6.91
Dyspnea	97.47	0.74	1.80

**Table 5:** Participants’ responses to preventive measures for SARS-CoV-2 infection.

Preventive Measures for SARS-CoV-2 Infection	True/%	False/%	Unclear/%
Frequent hand-washing	99.77	0.14	0.09
Keep a distance from people	99.72	0.18	0.09
Avoid rubbing eyes, mouth and nose	98.89	0.60	0.51
Wear a mask in public	99.95	0.05	0.00
Try not to touch elevator buttons, door handles and other public facilities directly	99.12	0.37	0.51
Ventilate the Room	99.36	0.32	0.32
Take antibiotics	14.37	72.96	12.67
Eat garlic	17.55	65.27	17.18
COVID-19 vaccination	95.12	0.69	4.19

**Table 6:** Frequency distribution and chi-squared analysis of intentions of COVID-19 vaccination under EUA.

Items	Willing to Vaccinate (%)	Reluctance to Vaccinate (%)	*χ* ^2^	*p*
Gender			32.06	<0.001
Male	76.8	23.2		
Female	65.4	34.6		
Age			26.76	<0.001
18–30	63.5	36.5		
31–40	70.6	29.4		
41–50	70.8	29.2		
≥51	79.7	20.3		
Region			1.52	0.218
Urban	69.2	30.8		
Rural	72.0	28.0		
Highest level of education			40.454	<0.001
Technical secondary school and below	85.8	14.2		
Junior College/University	69.0	31.0		
Master degree or above	62.4	37.6		
Monthly income/CNY			10.876	0.012
≤3000	66.2	33.8		
3001–5000	72.7	27.3		
5001–10,000	72.1	27.9		
>10,000	65.4	34.6		
Scores of COVID-19 related knowledge			15.47	<0.001
≥9	72.3	27.7		
[8, 9)	64.4	35.6		
<8	62.7	37.3		
Scores of COVID-19 transmission			2.66	0.265
≥9	69.4	30.6		
[8, 9)	71.1	28.9		
<8	67.4	32.6		
Scores of COVID-19 infection symptoms			0.647	0.724
≥9	69.5	30.5		
[8, 9)	70.2	29.8		
<8	71.9	28.1		
Scores of COVID-19 prevention measures			3.75	0.153
≥9	71.1	28.9		
[8, 9)	66.2	33.8		
<8	70.4	29.6		
You will be infected with SARS-CoV-2 this fall and winter			25.38	<0.001
Yes	87.3	12.7		
Uncertain	80.9	19.1		
No	68.4	31.6		
COVID-19 will break out again this year			24.53	<0.001
Yes	77.1	22.9		
uncertain	72.3	27.7		
No	66.1	33.9		
Views on the safety of COVID-19 vaccine			291.82	<0.001
Safe	88.7	11.3		
Uncertain	85.7	14.3		
Insecurity	53.7	46.3		
Views on the protective effect of COVID-19 vaccine			276.41	<0.001
Effective	89.6	10.4		
Uncertain	83.5	16.5		
Ineffective	54.2	45.8		

**Table 7:** Multivariate logistic regression analysis of influencing factors of the respondents’ willingness to vaccinate against COVID-19.

Characteristics	OR	95% CI	*p*-Value
Gender			
Female	Ref		
Male	1.54	1.24–1.92	<0.001
Highest level of education			
Technical secondary school and below	Ref		
Junior College/University	0.45	0.30–0.68	<0.001
Master degree or above	0.35	0.22–0.56	<0.001
Scores of COVID-19 related knowledge			
<8	Ref		
[8, 9)	0.98	0.68–1.44	0.929
≥9	1.46	1.06–2.00	0.020
Views on the safety of COVID-19 vaccine			
Insecurity	Ref		
Uncertain	3.17	2.32–4.33	<0.001
Safe	2.56	1.68–3.92	<0.001
Views on the protective effect of COVID-19 vaccine			
Ineffective	Ref		
Uncertain	2.45	1.81–3.31	<0.001
Effective	3.06	1.99–4.71	<0.001
I will be infected with SARS-CoV-2 this fall and winter			
No	Ref		
Uncertain	2.38	1.09–5.20	0.030
Yes	2.18	1.27–3.77	0.005

**Table 8:** Reasons for willingness or unwillingness to receive the COVID-19 vaccine once it is authorized under Emergency Use Administration (EUA) n (%).

Reasons	Disagree Strongly	Disagree	Neutral or Unknown	Agree	Agree Strongly
Reasons for willingness to receive COVID-19 vaccine					
COVID-19 vaccination is safe	19 (1.17)	12 (0.74)	238 (14.67)	538 (33.17)	815 (50.25)
Vaccination is very effective in preventing COVID-19	14 (0.86)	9 (0.55)	241 (14.86)	554 (34.16)	804 (49.57)
Can protect family/friends/colleagues from infection	22 (3.54)	22 (1.36)	176 (10.85)	471 (29.04)	931 (57.40)
Vaccination is beneficial if recommended by the country	18 (1.11)	7 (0.43)	120 (7.4)	465 (28.67)	1012 (62.39)
Reasons for reluctance to get the COVID-19 vaccine					
There are no COVID-19 cases in the region and no vaccinations are required	155 (2.63)	75 (13.66)	146 (26.59)	86 (15.66)	87 (15.85)
Doubting about the effectiveness of the COVID-19 vaccine	80 (14.58)	69 (12.57)	181 (32.97)	123 (22.40)	96 (17.49)
Worrying about the side effects of the COVID-19 vaccine	40 (7.29)	38 (6.92)	138 (25.14)	168 (30.60)	165 (30.05)
Community health service centers are not convenient for vaccination	263 (47.91)	105 (19.13)	111 (20.22)	37 (6.74)	33 (6.01)

## Data Availability

The datasets generated and analyzed during this study are not publicly available due to the institute’s data security and sharing policy, but are available from the corresponding author on reasonable request.
